# 

**Published:** 1898-12

**Authors:** 


					﻿Vol. XVIII.
DECEMBER, 1898.
NO. 12?
THE
jiomœopathic
A Monthly Journal of Homoeopathic Materia Medica
and Clinical Medicine.
“ He it a freeman whom truth makes free, and all are elaves beside."
"Seek the Truth: come whence it may, cost what it wilL."
EDITED AND PUBLISHED BY
WALTER M. JAMES, M. E>.
PHILADELPHIA :
TsTo. 1231 LOCUST STREET.
- Yearly Subscription, $2.50, invariably in advance. Single members, 25 ete.
Entered at the Philadelphia Post-Office at 8eoond*Clasa Mail Matter.
				

